# Supplementary material for: Char dominates black carbon aerosol emission and its historic reduction in China
Source: Nat Commun. 2023 Oct 13;14:6444. doi: 10.1038/s41467-023-42192-8 (PMC10575950; doi:10.1038/s41467-023-42192-8)
Supplement: Supplementary file 1 — Supplementary Information [file 41467_2023_42192_MOESM1_ESM.pdf]

Supporting Information for

Char Dominates Black Carbon Aerosol Emission and Its Historic Reduction in China

Junjie Cai[a,1], Hongxing Jiang[a,1], Yingjun Chen[a, b*], Zeyu Liu[a], Yong Han[a], Huizhong Shen[c], Jianzhong Song[d, e], Jun Li[d, e], Yanlin Zhang[f, g], Rong Wang[a], Jianmin Chen[a], Gan Zhang[d, e*]

[a] Shanghai Key Laboratory of Atmospheric Particle Pollution and Prevention (LAP[3]), Department of Environmental Science and Engineering, Fudan University, Shanghai 200438, China

[b] Shanghai Institute of Pollution Control and Ecological Security, Shanghai 200092, China

[c] Guangdong Provincial Observation and Research Station for Coastal Atmosphere and Climate of the Greater Bay Area, School of Environmental Science and Engineering, Southern University of Science and Technology, Shenzhen, 518055, China

[d] State Key Laboratory of Organic Geochemistry, Guangzhou Institute of Geochemistry, Chinese Academy of Sciences, Guangzhou, 510640, China

[e] Guangdong-Hong Kong-Macao Joint Laboratory for Environmental Pollution and Control, Guangzhou Institute of Geochemistry, Chinese Academy of Science, Guangzhou 510640, China

[f] Yale–NUIST Center on Atmospheric Environment, International Joint Laboratory on Climate and Environment Change (ILCEC), Nanjing University of Information Science and Technology, Nanjing, 210044, China

[g] Jiangsu Provincial Key Laboratory of Agricultural Meteorology, College of Applied Meteorology, Nanjing University of Information Science and Technology, Nanjing, 210044, China

[1]Junjie Cai and [1]Hongxing Jiang contributed equally to this work.

[*]Corresponding authors: yjchenfd@fudan.edu.cn (Y. Chen); zhanggan@gig.ac.cn (G. Zhang)

Contents of this file

Text S1 Sampling details

Text S2 Calculation of emission factors (EFs) and modified combustion efficiency (MCE)

Text S3 Weighted average method

Table S1 Mass-based emission factors (EFs) and char to soot ratio ($R_{C/S}$) from different BC emission sectors

Table S2 Comparison of emission factors (EFs) of black carbon (BC) between previous studies and our study

Table S3 Comparison of emission factors (EFs) of char to soot ratio ($R_{C/S}$) between our study and previous studies

Table S4 Annual emission of black carbon (BC), char and soot in China (Unit: Gg) from 1960-2017 based on the latest emission inventory

Table S5 Technical Parameters of the Test diesel vehicles

Table S6 Technical Parameters of the Test gasoline vehicles

Table S7 Technical Parameters of the Test forklift vehicles

Table S8 Technical Parameters of the Test Ocean going vessels

Figure S1 Historical data of emission factors (EF) of black carbon (BC) vs. measured data in this manuscript

Figure S2 Comparison of char (a), soot (b) emission and char to soot ratio (c) in China between atmospheric and sediment cores

Figure S3 Emission factors (EF) and char to soot ratio of modified residential fuel combustion under

different chamber temperatures

Figure S4 Sketch of the combustion and dilution sampling system of field measurements and Quartz tube furnace

Text S1. Sampling details

We conducted stove-top field measurements of RS in 122 rural households in 10 eastern provinces (Shanghai, Jiangsu, Zhejiang, Anhui, Hubei, Shandong, Hebei, Jilin, Heilongjiang, Neimeggu) from September 2018 to July 2022. To reflect the real combustion situation in rural areas, representative fuels and stoves were used in the combustion measurements based on the daily cooking and heating habits of residents. Solid fuels were divided into biomass (straw and firewood) and coal. Straw is divided into nine categories depending on their type (corn, corn cobs, soybeans, rice, cotton, sorghum, reeds, peanuts, and bamboo), firewood is divided into branches and trunks depending on the part selected for burning, while coal is divided into chunk coal and honeycomb coal depending on its treatment process. Biomass is burned uniformly in brick stoves, while chunk coal and honeycomb coal are burned in separate stoves (traditional iron stove (TIS), improved iron stove (IIS) and honeycomb briquette stove (HBS)). Brick stoves is about 50 cm high and equipped with 1-3 iron pots. TIS and IIS are mainly used for coal heating. IIS has a larger internal space than TIS. In addition, the side windows of the IIS can be opened for fuel and air supply to achieve higher combustion efficiency. HBS use only honeycomb briquettes as heating fuel. They are about 40-60 cm high and have a cylinder chamber in the middle which can burn 3-4 honeycomb briquettes. The exact shape and photos of the stoves can be found in the previous articles of our group(*1*). Flue gases emitted from the stack are sampled and measured using a mobile dilution sampling system consisting of a dilution channel, two

flue gas analyzers (GA21plus, Madur, Austria) and sampling instruments (Fig. S2). A portion of the flue gas emitted from the stack is drawn into the dilution channel and mixed with clean air filtered with polypropylene fibers of 1 μm pore size for cooling and dilution. The pre and post dilution CO and CO_2 concentrations are measured online by two flue gas analyzers to obtain the actual dilution ratio, which is 10-20 in the sampling.

Two different references were consulted for the classification and sampling of on-road vehicles and construction machinery respectively(*2, 3*). The actual emissions of gasoline and diesel vehicles with different emission standards (China-III, China-IV, China-V and China-VI) under different driving conditions (idling speed; suburban main road driving; high speed road driving) were collected between December 2020 and March 2022 using follow-up sampling (detailed in Tables S5 and S6). At the same time, particulate matter was collected from the exhaust of 6 forklift trucks of different tonnages and aftertreatment technology levels (with or without diesel particulate filter (DPF)) under different driving conditions (idling, unloaded and loaded) during January-December 2021 using the same sampling method, detailed in Table S7. Specifically, we combined a vehicle emission sampling system to collect particulate matter emissions. The equipment is mounted on a separate sampling vehicle for each sampling session, and the sampling vehicle follows the subject vehicle at all times. The equipment is powered by a generator on the sampling vehicle to avoid overloading the engine. A homemade dilution sampling system was used to control the dilution and sampling flow

rate between the sampling vehicle and the test workshop (Figure S3). The pre-dilution and post-dilution exhaust gases were monitored at all times by two GA21 exhaust gas analyzers and flow meters to determine the instantaneous dilution ratio. A vacuum pump with active flow control is used at the end of the post-dilution sampling channel to ensure a flow rate of 40 L/min through the quartz membrane.

A total of 76 particle samples were collected from three ocean-going vessels (OGV, an Aframax oil tanker, a Newcastlemax bulk carrier and a Capesize bulk carrier, detailed parameters in Table S8) between December 2020 and August 2021(*4*). All samples were collected under steady-state navigation of the OGV, and the corresponding sailing parameters including: engine load (25%, 50%, 75%, 100%), engine power rating (different power of main engine and generator) and fuel type (Marine gas oil and Heavy fuel oil). Specific sampling and dilution system is built with reference to(*5, 6*).

The real-world measurement of ICB and PCB were conducted in Hebi City, Henan Province, from 1 to 5 November 2018. Hebi City is one of the major air pollution transmission channels in the Beijing-Tianjin-Hebei region ("2+26" cities). In summary, for ICB, we collected emission samples from a coal-fired boiler, two coking plants, and two pharmaceutical factories in the vicinity of Hebi City, based on the local industrial source emission inventory provided by the Hebi Environmental Protection Bureau. As for power plant sources, we selected two representative local biomass power plants and a coal-fired power plant for sampling. The samplers were installed at the flue gas outlet after the dust removal.

To further illustrate the formation mechanism and the influence of combustion condition on char and soot formation in RS, laboratory combustion experiments of biomass and coal were conducted through multiple combustion conditions. The quartz tube furnace sampling is done with a self-built quartz tube furnace combustion system, which consists of four parts: air supply module, combustion control module, flue gas dilution module, and sampling module (Fig S3). For the specific structure, please refer to the literature(*7*). Six solid fuels, including rice straw, corn straw, wheat straw, pine wood, poplar wood and Xuzhou coal (from Xuzhou City, Jiangsu Province, China) were used quartz tube furnace combustion. For each experiment, 1 g of dry fuel powder (60-80 mesh) was burned at different ignition temperatures (defined as furnace temperatures: 300, 400, 500, 600, 700, 800 and 900°C) with different oxygen supply conditions (10.5% vs. 21% O_2). Before each combustion experiment, the furnace was heated to the setting temperature. A 1g fuel sample was weighed and placed onto the ceramic crucible. The ceramic crucible was first hung in the low temperature zone, and then was lifted to the combustion zone. The high-purity of compressed air (flow of 5 L/min) was let into the quartz tube from the bottom, and then flue gas rose through the quartz tube and entered the dilution sampling system. The diluted flue gas was subsequently introduced to a filter sampler (quartz fiber filter; d = 90 mm) to collect the total suspended particles. Each combustion experiment was repeated twice for all fuel/temperature combinations to collect samples.

Text S2. Calculation of emission factors (EFs) and modified combustion efficiency (MCE)

EFs were calculated by dividing the emission by the mass of the fuel consumed, and expressed as grams of emission per kilogram of consumed dry fuel (g·kg^{-1})(*8*). For particulate pollutants (i.e. OC, BC, Char and Soot), the EFs were calculated as:

$$EE_{P=}\frac{m_{fiiter}}{Q}\frac{V_{Total-chimney}}{M_{fuel}}DR$$

Where EE_p is the EF of particulate pollutants for the specific crop residue; m_{fiiter} is the mass of pollutants collected on the filter; $V_{Total-chimney}$ is the total volume of exhaust flowing through the chimney during the experiment (m^3) at standard temperature and pressure; Q is the sampling volume through the filter (m^3) at standard temperature and pressure; M_{fuel} is the mass of burned fuel (kg, dry basis); and DR is the dilution ratio in the dilution sampler, which was determined using the measured stack, diluted, and background CO_2 concentrations (i.e. CO_2,Stk, CO_2,Dil and CO_2,Bkg, respectively), where:

$$DR=\frac{CO_{2,Stk}-CO_{2,Bkg}}{CO_{2,Dil}-CO_{2,Bkg}}$$

For CO_2 and CO, the EFs were calculated using online monitored concentrations as follows:

$$EF_X=\frac{V_{Total-Chimney}}{m_{fuel}}\frac{C_{x,Dilute}}{V_x}M_XDR$$

where $C_{x,Dilute}$ is the average concentration (molar fraction) measured in the dilution sampler; V_x is the molar volume of gas at standard temperature and pressure (0.0224 m^3) and M_x is the molecular weight of species x (g·mol^{-1}).

When CO_2 and CO were measured, the MCE was reported as:

$$MCE = \frac{\Delta[CO_2]}{\Delta[CO] + \Delta[CO_2]}$$

Where $\Delta[CO_2]$ and $\Delta[CO]$ are the excess molar mixing ratios of CO_2 and CO, respectively.

Text S3. Weighted average method

It is necessary to normalize the EF_{EC} and $R_{C/S}$ of the broad categories of sources from the measurements in this study in order to calculate the EF_{EC} and $R_{S/C}$ of the population of individual sources. For biomass, five types of straws that account for more than 90% of total straw burning in China (17.5% for rice, 19.5% for wheat, 39.1% for corn, 5.4% for beans, and 9.3% for oil crops) were combined into straw using their weighted average results overall results(*9*). The total fuel use ratio of firewood to straw was about 1.2:1, and the overall result of combining straw and firewood into biomass was this weighted result(*10*). A weighted average result for coal was obtained by considering a total fuel usage ratio of lump coal/briquette coal of 4:1(*11, 12*). For the three driving states of the motor vehicle, the results of high speed: normal speed: idle speed = 5:4:1 are weighted and averaged, and the mathematical average method is adopted in gasoline and diesel workshops with different emission standards(*13*). For OGVs, both the generator and the main engine are kept on throughout the journey, and the usage of heavy fuel oil (HFO) is about 4 times that of marine gas oil (MGO). Therefore, the ship emissions are weighted with the result of HFO: MGO = 4:1(*5*).

Table S1 Mass-based emission factors (EFs) and char to soot ratio ($R_{C/S}$) from different emission sectors

Stove/Oil type	Fuel type/Emission Standard	Sample number	EF_{OC} (g/kg)	EF_{EC} (g/kg)	EF_{Char} (g/kg)	EF_{Soot} (g/kg)	Char/Soot
BS	Bamboo	3	3.72 ± 0.67	3.33 ± 0.68	2.81 ± 0.76	0.52 ± 0.08	5.64 ± 2.43
BS	Bulrush	2	3.90 ± 0.90	2.24 ± 0.58	1.88 ± 0.45	0.36 ± 0.13	5.35 ± 0.71
BS	Peanut	4	15.56 ± 2.46	2.06 ± 0.68	1.54 ± 0.51	0.52 ± 0.18	4.98 ± 0.03
BS	Soybean	10	4.05 ± 3.30	1.37 ± 0.92	1.12 ± 0.83	0.25 ± 0.19	5.87 ± 3.15
BS	Maize	13	2.84 ± 0.96	1.00 ± 0.59	0.78 ± 0.49	0.22 ± 0.15	4.16 ± 2.68
BS	Cotton	2	2.25 ± 1.53	0.93 ± 0.70	0.75 ± 0.59	0.18 ± 0.11	3.87 ± 0.83
BS	Corncob	8	4.73 ± 3.73	0.86 ± 0.14	0.65 ± 0.19	0.21 ± 0.12	4.38 ± 3.4
BS	Rice	4	2.24 ± 0.97	0.73 ± 0.46	0.60 ± 0.38	0.13 ± 0.08	5.26 ± 4.75
BS	Sorghum	3	1.27 ± 0.60	0.59 ± 0.13	0.42 ± 0.07	0.17 ± 0.06	2.63 ± 0.49
Crop residue Burning		49	3.93 ± 2.71	0.97 ± 0.57	0.76 ± 0.49	0.22 ± 0.15	4.6 ± 3.61
BS	Logwood	18	4.81 ± 3.09	0.83 ± 0.48	0.67 ± 0.42	0.16 ± 0.10	4.51 ± 2.28
BS	Brushwood	17	5.35 ± 7.20	1.21 ± 0.86	0.99 ± 0.75	0.22 ± 0.16	5.42 ± 3.42
Wood Burning		35	5.08 ± 5.15	1.02 ± 0.67	0.83 ± 0.59	0.19 ± 0.13	4.97 ± 2.85
Residential Biomass Burning		84	4.70 ± 4.33	1.00 ± 0.64	0.81 ± 0.56	0.20 ± 0.14	4.78 ± 3.22
BS	Chunk-BS	4	13.46 ± 10.86	1.22 ± 0.16	0.63 ± 0.13	0.59 ± 0.16	1.17 ± 0.54
TIS	Chunk-TIS	14	4.13 ± 1.50	0.83 ± 0.53	0.54 ± 0.45	0.29 ± 0.25	1.89 ± 1.40
IIS	Chunk-IIS	13	1.88 ± 2.87	0.13 ± 0.15	0.08 ± 0.08	0.05 ± 0.07	2.14 ± 2.36
Chunk Coal Combustion		31	6.49 ± 5.08	0.73 ± 0.28	0.42 ± 0.22	0.31 ± 0.16	1.73 ± 1.44
HBS	Briquette	7	0.32 ± 0.19	0.07 ± 0.04	0.05 ± 0.03	0.02 ± 0.01	3.56 ± 1.43
Residential Coal Combustion		38	5.25 ± 4.10	0.60 ± 0.23	0.35 ± 0.18	0.25 ± 0.13	2.08 ± 1.82
0#	China-III	3	0.59 ± 0.37	0.16 ± 0.08	0.11 ± 0.05	0.05 ± 0.03	2.46 ± 0.65
	China-IV	3	0.12 ± 0.02	0.07 ± 0.04	0.04 ± 0.02	0.03 ± 0.02	1.04 ± 0.01
	China-V	9	0.08 ± 0.02	0.11 ± 0.05	0.05 ± 0.02	0.06 ± 0.03	1.09 ± 0.34

Stove/Oil type	Fuel type/Emission Standard	Sample number	EF_{OC} (g/kg)	EF_{EC} (g/kg)	EF_{Char} (g/kg)	EF_{Soot} (g/kg)	Char/Soot
0#	China-VI	12	0.05 ± 0.02	0.02 ± 0.00	0.005 ± 0.002	0.01 ± 0.004	0.45 ± 0.07
	Diesel Vehicle	27	0.21 ± 0.11	0.09 ± 0.04	0.05 ± 0.02	0.04 ± 0.02	1.16 ± 0.95
#E93	China-IV [a]	18	0.16 ± 0.08	0.017 ± 0.015	0.012 ± 0.014	0.005 ± 0.002	2.46 ± 2.63
	China-V [a]	18	0.08 ± 0.04	0.013 ± 0.007	0.006 ± 0.004	0.007 ± 0.004	0.82 ± 0.50
	Gasoline Vehicle	36	0.12 ± 0.06	0.015 ± 0.011	0.009 ± 0.009	0.006 ± 0.003	1.44 ± 1.27
On-Road Vehicle		63	0.17 ± 0.09	0.05 ± 0.03	0.03 ± 0.01	0.02 ± 0.01	1.14 ± 0.92
0#	Forklift-Without DPF	18	0.68 ± 0.65	0.59 ± 0.31	0.36 ± 0.24	0.23 ± 0.16	1.77 ± 1.07
	Forklift-With DPF	18	0.08 ± 0.04	0.011 ± 0.003	0.011 ± 0.003	0.009 ± 0.003	1.22 ± 0.35
HFO	Main Engine	25	1.12 ± 0.60	0.054 ± 0.038	0.027 ± 0.019	0.027 ± 0.022	1.15 ± 0.57
	Generator	15	0.47 ± 0.32	0.147 ± 0.082	0.106 ± 0.069	0.041 ± 0.024	3.26 ± 2.81
MGO	Main Engine	15	0.51 ± 0.38	0.035 ± 0.030	0.013 ± 0.011	0.024 ± 0.02	0.58 ± 0.24
	Generator	6	0.28 ± 0.24	0.113 ± 0.068	0.07 ± 0.05	0.04 ± 0.02	1.67 ± 0.92
Ocean-Going Vessels		61	0.60 ± 0.39	0.09 ± 0.05	0.05 ± 0.04	0.03 ± 0.02	1.99 ± 1.48
Non-road Vehicle		97	0.64 ± 0.52	0.34 ± 0.18	0.21 ± 0.14	0.13 ± 0.09	1.39 ± 0.60
Coke ovens		2	0.65 ± 0.24	0.80 ± 0.96	0.11 ± 0.13	0.69 ± 0.83	0.16 ± 0.01
Brick kilns		1	1.3	0.27	0.11	0.16	0.68
Pharmaceutical plants		6	5.15 ± 1.70	1.52 ± 1.48	0.44 ± 0.36	1.10 ± 1.13	0.46 ± 0.27
Industrial emission		9	2.36 ± 0.80	0.86 ± 0.82	0.22 ± 0.23	0.64 ± 0.11	0.49 ± 0.40
Power plant		3	1.48 ± 1.44	0.11 ± 0.12	0.03 ± 0.03	0.09 ± 0.08	0.42 ± 0.15

Table S2 Comparison of emission factors (EFs) of black carbon (BC) between previous studies and our study

EF_{BC} (g/kg)	Year	Fuel type	Burning methods	Reference
0.09 - 3.29	2009	6 straws and 2 woods	Kang	(*14*)
0.88 ± 0.6	2013	Crop residue	Biomass stove	(*15*)
1.5 ± 0.72	2013	Firewood	Biomass stove	(*15*)
0.59 ± 0.49	2013	Branch	Biomass stove	(*15*)
0.42 - 2.70	2014	4 straws	1 year-15 year Biomass stove	(*16*)
0.28 ± 0.14	2015	Woods	Biomass stove	(*17*)
0.40 ± 0.27	2017	Woods	Wood gasifier stove	(*18*)
0.73 ± 2.8	2018	3 straws and 2 woods	Brick Biomass stove	(*19*)
0.42 ± 1.01	2018	9 kinds of crop residues	Biomass stove	(*11*)
0.74 ± 0.74	2021	Crop-residues	Biomass stove	(*20*)
0.97 ± 0.57	2022	11 kinds of crop residues	Biomass stove	this study
1.02 ± 0.67	2022	Logwood and brushwood	Biomass stove	this study
0.064 - 0.675	2005	Bituminous coal	Coal stove	(*21*)
0.017	2005	Anthracite	Coal stove	(*21*)
0.99 - 13.25	2009	Chunk coal LVB-HVB	Coal stove	(*22*)
0.043 - 0.25	2009	Honeycomb-coal LVB-HVB	Coal stove	(*22*)
1.48 - 23.5	2008	Chunk bituminous coal	Hec Coal stove	(*23*)
0.009 - 0.180	2008	Honeycomb bituminous coal	Heb Coal stove	(*23*)
0.004 - 0.04	2013	Briquettes	Improved stove with a chimney	(*15*)
0.022 - 0.052	2013	Coal cake	Brick stove with a flue	(*15*)
0.0036 ± 0.0024	2014	Anthracite briquette coal	Coal stove	(*24*)
0.18 ± 0.21	2014	Bituminous briquette coal	Coal stove	(*24*)
0.019 ± 0.016	2014	Anthracite coal chunk	Coal stove	(*24*)
EFBC (g/kg)	Year	Fuel type	Burning methods	Referen ce
4.8 ± 6.2	2014	Bituminous coal chunk	Coal stove	(*24*)

2.04 ± 1.23	2015	Chunk bituminous coal	Hec Coal stove	(*12*)
0.83 ± 0.71	2015	Honeycomb bituminous coal	Heb Coal stove	(*12*)
1.73 - 13.41	2018	Chunk	SC, HD and LW Stove	(*11*)
0.21 - 0.80	2018	Briquettes	WJ Stove	(*11*)
0.42 ± 0.33	2021	Chunk-Coal	Coal stove	(*20*)
0.73 ± 0.28	2022	Chunk-Coal	BS, TIS, IIS	this study
0.07 ± 0.04	2022	Briquettes	HES Coal stove	this study
0.00076 - 0.0241	2013	Gasoline	On-road-vehicle	(*25*)
0.0681-7.20(0.234)	2014	Gasoline-diesel	On-road-vehicle	(*26*) and 59 other articles
0.09 ± 0.04	2022	Diesel	On-road-vehicle	this study
0.015 ± 0.011	2022	Gasoline	On-road-vehicle	this study
0.19 ± 0.04 - 0.64 ± 0.38	2016	MGO	Ocean going vessels	(*27*)
0.07 - 6.41	2019	MGO	Offshore area boats	(*28*)
0.09±0.01 - 0.15 ± 0.02	2021	MGO-HFO	Ocean going vessels	(*29*)
0.09 ± 0.05	2022	MGO-HFO	Ocean going vessels (Main engine and generator)	this study
0.09 - 2.92	2020	Diesel	Non-road vehicle	(*3*)
0.011 ± 0.003 - 0.59 ± 0.31	2022	Diesel	forklift	this study

Table S3. Comparison of emission factors (EFs) of char to soot ratio (RC/S) between our study and previous studies

Country	Year	Char/Soot	Sector	Fuel type	Reference
U.S.	1999	25.00	Open burning	Biomass	(*30*)
U.S.	1999	0.35	RS	Pine fence	(*30*)
U.S.	1999	33.33	RS	Mesquite burn	(*30*)
U.S.	1999	16.67	RS	Tamarisk burn	(*30*)
U.S.	1999	33.33	RS	Huisache burn	(*30*)
U.S.	1999	12.50	RS	Grass burn	(*30*)
U.S.	1999	0.98	RS	Biomass	(*30*)
U.S.	1999	2.13	RS	Smoked chicken	(*30*)
U.S.	1999	3.85	RS	Charbroiled chicken	(*30*)
U.S.	1999	4.35	RS	Chicken over propane	(*30*)
U.S.	1999	5.26	RS	Charbroiled Hamburger	(*30*)
U.S.	1999	0.66	RS	Steak Stir-fry	(*30*)
U.S.	2003	0.15	RS	Ponderosa pine wood	(*30, 31*)
U.S.	2003	25.00	RS	Ponderosa pine needles	(*30, 31*)
U.S.	2003	4.35	RS	White pine needles	(*30, 31*)
U.S.	2003	10.00	RS	Excelsior	(*30, 31*)
U.S.	2003	33.33	RS	Sagebrush	(*30, 31*)
U.S.	2003	0.45	RS	Dumbo grass	(*30, 31*)
China	2003	11.11	RS	Biomass burning	(*32*)
China	2004	4.35	RS	Biomass	(*33*)
China	2012	7.14	RS	Biofuel burning	(*34*)
China	2022	4.60 ± 3.61	RS	Crop residues	this study
China	2022	4.97 ± 2.85	RS	Firewood	this study
China	2021	1.39	RS	Bituminous Coal	(*34*)
China	2022	2.08±1.82	RS	Chunk and briquettes	this study
U.S.	1999	0.43	VE	Road side	(*30*)
China	2002	0.30	VE	Diesel exhausts (road side)	(*35*)
China	2002	0.65	VE	LPG exhausts (road side)	(*35*)
China	2002	0.70	VE	Gasoline exhausts (road side)	(*35*)
China	2022	1.44 ± 1.27	VE	Gasoline exhausts	this study
China	2022	1.16 ± 0.95	VE	Diesel exhausts	this study
China	2022	1.22 ± 0.35	VE	Forklift exhausts	this study
China	2022	1.99 ± 1.48	VE	Ocean-going-Vessels exhausts	this study
U.S.	1999	0.40	CB	Aluminum plant	(*30*)
U.S.	1999	0.86	CB	Coal fly ash	(*30*)
U.S.	1999	0.96	CB	Cement kiln	(*30*)
China	2022	0.49 ± 0.40	CB	Coking	this study
China	2022	0.42 ± 0.15	CB	Coal combustion	this study

Table S4. Annual emission of black carbon (BC), char and soot in China (Unit: Gg) from 1960-2017 based on the latest emission inventory

Year	Soot	Soot Uncertainty	Char	Char Uncertainty	Total BC	Char / Soot	C/S Uncertainty
1960	486.30	90.62	1054.16	196.63	1540.46	2.17	0.81
1961	359.34	74.56	985.25	204.73	1344.59	2.74	1.14
1962	301.18	66.73	962.98	213.55	1264.16	3.20	1.42
1963	300.15	67.12	984.38	220.38	1284.53	3.28	1.47
1964	309.67	69.06	1011.23	225.84	1320.90	3.27	1.46
1965	329.29	72.51	1042.62	229.95	1371.91	3.17	1.4
1966	351.23	76.11	1067.44	231.58	1418.67	3.04	1.32
1967	331.20	73.71	1070.98	238.44	1402.18	3.23	1.44
1968	333.34	74.11	1080.39	240.47	1413.73	3.24	1.44
1969	373.09	80.37	1122.51	242.17	1495.60	3.01	1.3
1970	416.25	86.99	1165.16	243.77	1581.41	2.80	1.17
1971	457.20	92.99	1198.37	243.77	1655.57	2.62	1.07
1972	485.57	96.96	1228.40	245.51	1713.97	2.53	1.01
1973	499.57	99.18	1250.38	248.47	1749.95	2.50	0.99
1974	490.45	98.42	1251.82	251.23	1742.27	2.55	1.02
1975	529.67	103.44	1279.63	250.10	1809.30	2.42	0.94
1976	534.19	104.39	1287.65	251.68	1821.85	2.41	0.94
1977	563.70	108.10	1310.93	251.60	1874.63	2.33	0.89
1978	603.25	112.78	1330.48	248.88	1933.73	2.21	0.82
1979	605.53	112.98	1332.38	248.76	1937.91	2.20	0.82
1980	606.37	113.65	1347.33	252.74	1953.70	2.22	0.83
1981	593.74	111.26	1319.88	247.58	1913.62	2.22	0.83
1982	600.16	110.73	1295.47	239.31	1895.63	2.16	0.8
1983	618.72	111.70	1275.09	230.36	1893.81	2.06	0.74
1984	667.93	116.53	1285.18	224.35	1953.11	1.92	0.67
1986	715.91	116.86	1210.42	197.74	1926.33	1.69	0.61
1987	774.86	121.90	1220.33	192.24	1995.19	1.57	0.55
1988	819.27	125.70	1219.62	187.29	2038.89	1.49	0.5
1989	850.61	127.82	1205.62	181.28	2056.23	1.42	0.46
1990	650.77	96.38	1432.29	212.39	2083.06	2.20	0.43
1991	644.85	94.99	1406.90	207.46	2051.75	2.18	0.65
1992	669.94	96.61	1413.10	203.87	2083.04	2.11	0.64
1993	765.18	106.72	1516.83	211.64	2282.01	1.98	0.61
1994	822.73	113.09	1570.07	215.97	2392.80	1.91	0.55
1995	922.37	126.31	1686.36	231.02	2608.73	1.83	0.52
1996	898.19	120.59	1637.06	220.12	2535.25	1.82	0.5
1997	874.48	117.04	1598.30	215.04	2472.78	1.83	0.49
1998	709.74	94.70	1380.40	186.13	2090.14	1.94	0.49
1999	586.39	80.30	1193.03	164.65	1779.42	2.03	0.52
2000	530.10	71.22	1093.40	147.89	1623.50	2.06	0.56
2001	648.41	88.23	890.07	121.60	1538.48	1.37	0.56

2002	597.77	82.30	849.15	117.22	1446.92	1.42	0.37
2003	602.28	79.92	848.05	113.44	1450.33	1.41	0.39
2004	581.80	76.00	831.47	109.67	1413.27	1.43	0.38
2005	617.63	79.50	841.68	108.88	1459.31	1.36	0.38
2006	616.83	78.21	830.79	105.92	1447.62	1.35	0.35
2007	620.89	76.76	810.76	100.79	1431.65	1.31	0.34
2008	612.96	74.85	795.43	97.81	1408.39	1.30	0.32
2009	692.21	82.37	829.57	99.38	1521.78	1.20	0.32
2010	704.22	82.86	833.86	98.69	1538.08	1.18	0.29
2011	704.90	82.38	830.60	97.56	1535.50	1.18	0.28
2012	686.17	79.98	795.73	93.11	1481.90	1.16	0.28
2013	716.89	82.17	775.81	89.31	1492.70	1.08	0.27
2014	704.27	79.91	750.43	85.59	1454.70	1.07	0.25
2015	653.84	74.08	701.86	79.84	1355.70	1.07	0.24
2016	629.90	71.04	665.50	75.36	1295.40	1.06	0.24
2017	604.13	67.88	625.37	70.57	1229.50	1.04	0.24

Table S5. Technical Parameters of the Test Diesel Vehicles

Car number	Manufacturers	Emission Standard	Year of manufacture	Vehicle weight (kg)	Driven distance (km)
1	Haowo	China-V	2020.4	4495	95148
2	JAC	China-III	2012.8	4290	117773
3	Foton	China-VI	2020.7	4495	21473
4	Dongfeng	China-IV	2016.4	4370	101961
5	Dongfeng	China-V	2020.6	4495	72787
6	JAC	China-V	2021.1	4495	6253
7	JAC	China-VI	2020.11	4495	83081
8	JAC	China-III	2011.4	4300	298892
9	JMC	China-VI	2021.2	4495	40357

Table S6 Technical Parameters of the Test Gasoline Vehicles

Car number	Manufacturers	Emission Standard	Vehicle weight (kg)	Power rating
1	Chang-an	China-V	1185Kg	76.5KW
2	Foton	China-IV	1180Kg	45/43.5KW
3	Chang-an	China-IV	995Kg	46.5KW
4	Foton	China-V	1619Kg	82/77KW

Table S7 Technical Parameters of the Test Forklift Vehicles

Car number	Manufacturers	Engine model	Net power/ton
1	Hang Cha	CPC35-AG51	3500kg
2	Hang Cha	CPC35N-RG51	3500kg
3	Hang Cha	CPC35N-RG51	3500kg
4	Heli	CPCWG038798	12000kg
5	Heli	CA6DF3-16GAH3U	12000kg
6	Heli	CA6DF3-16GAG3U	12000kg

Table S8 Technical Parameters of the Test Ocean Going Vessels

Type	Aframaxtype Oil Tanker	Newcastlemax Bulk Carriers	Capesize Bulk Carrier
dead weight	109,000 t	209,000 t	179,000 t
Length × Breadth × Depth (m)	249.9×44.0×21.2	299.9×50.0×25.2	292.0×45.0×24.9
Main engine	two-stroke low-speed	two-stroke low-speed	two-stroke low-speed
Power & speed	13500 kW, 91.1 rpm	15298 kW, 73 rpm	15748 kW, 75 rpm
Bore × Stroke	700 mm×3256 mm	700 mm×3256 mm	630 mm×2890 mm
Auxiliary engine	four-stroke medium-speed	four-stroke medium-speed	four-stroke medium-speed
Power & speed	1280 kW, 900 rpm	1180 kW, 900 rpm	900 kW, 900 rpm
Bore × Stroke	200 mm×300 mm	220 mm×320 mm	150 mm×250 mm

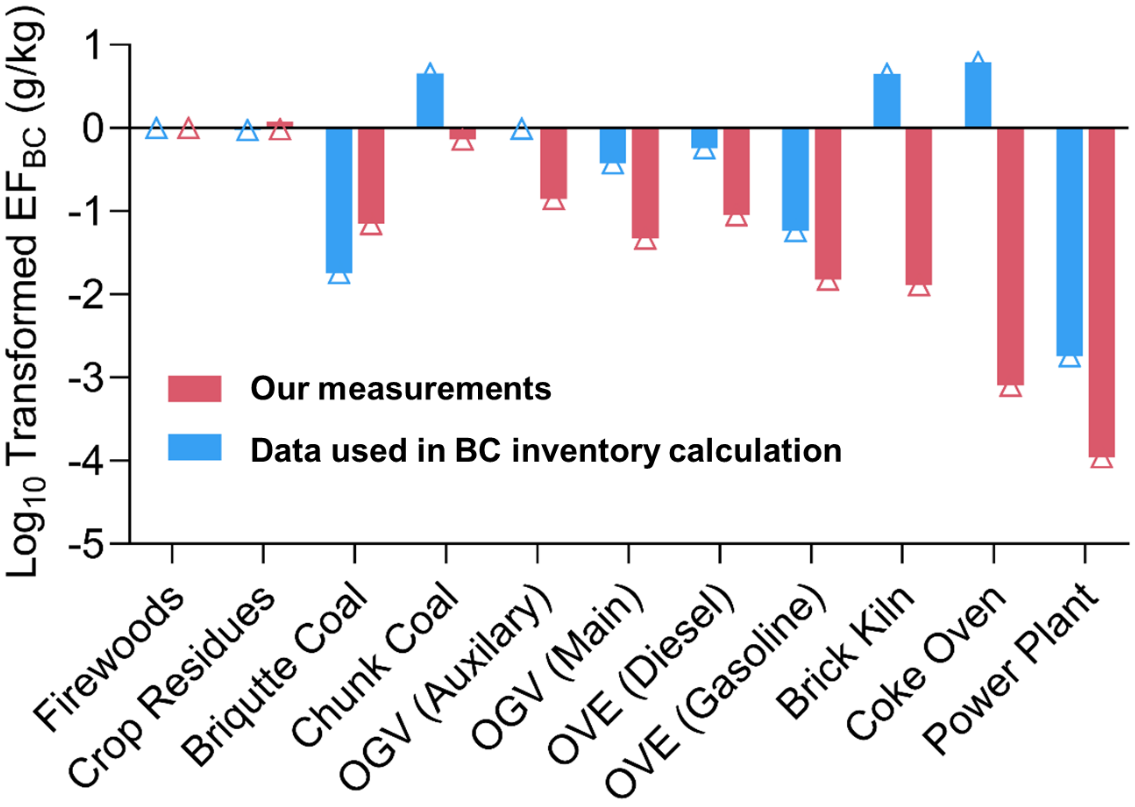

Figure S1. Historical data of emission factors (EF) of BC (*36, 37*) vs. measured data in this manuscript

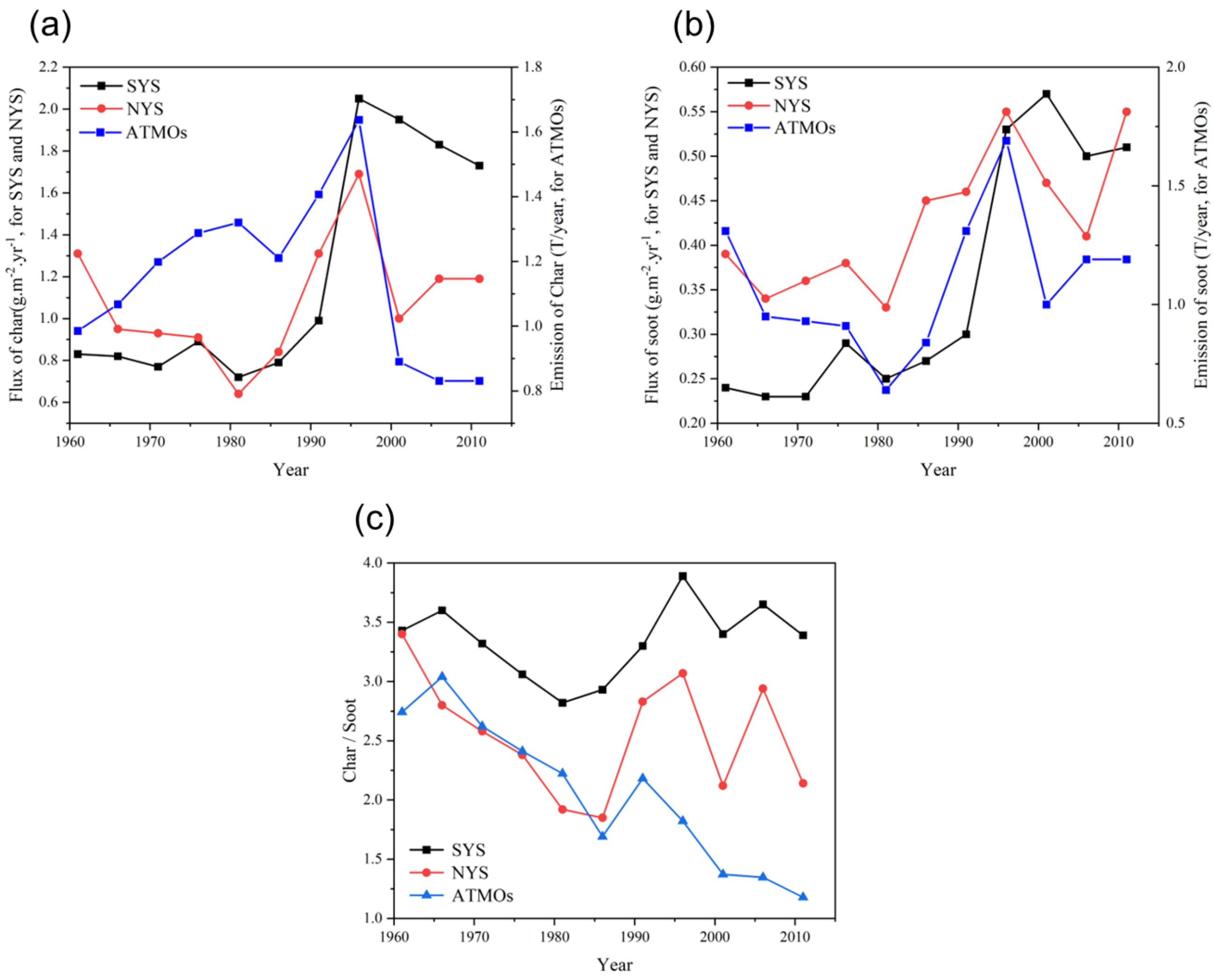

Figure S2. Comparison of char (a), soot (b) emission and char / soot (c) in China between atmospheric and sediment cores（South yellow sea: SYS, North yellow sea: NYS, Atmospheric annual emission: ATMOs）, detailed information of the sediment cores could be found in Fang et al(*38*).

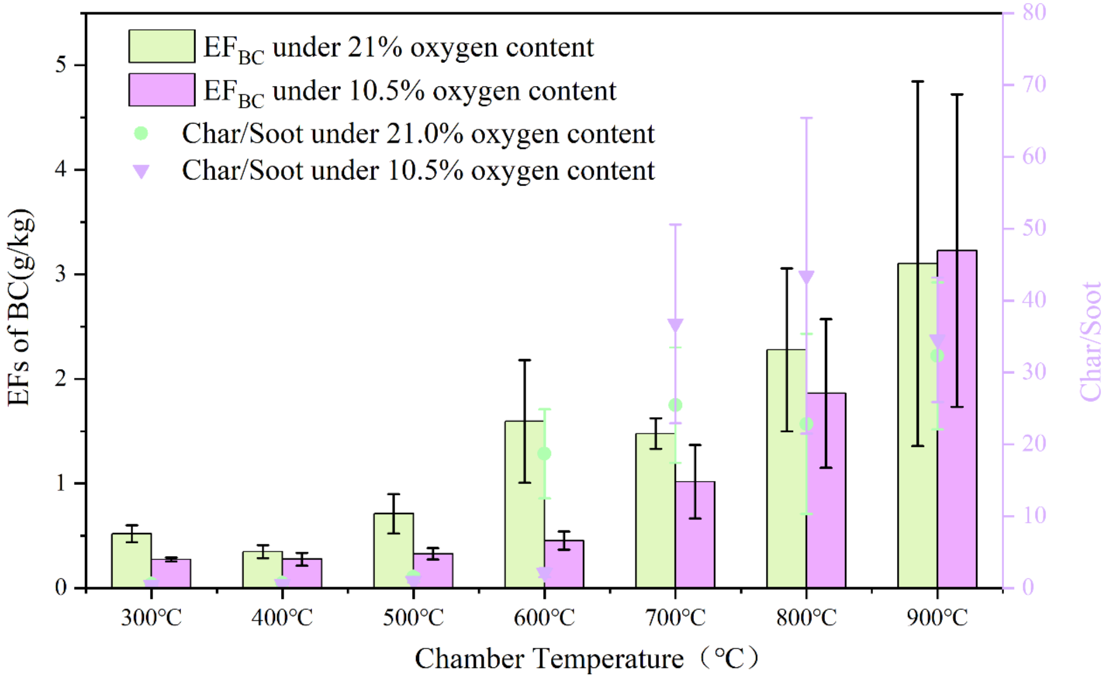

Figure S3 Emission factors (EF) and char to soot ratio of modified residential fuel combustion under different chamber temperatures

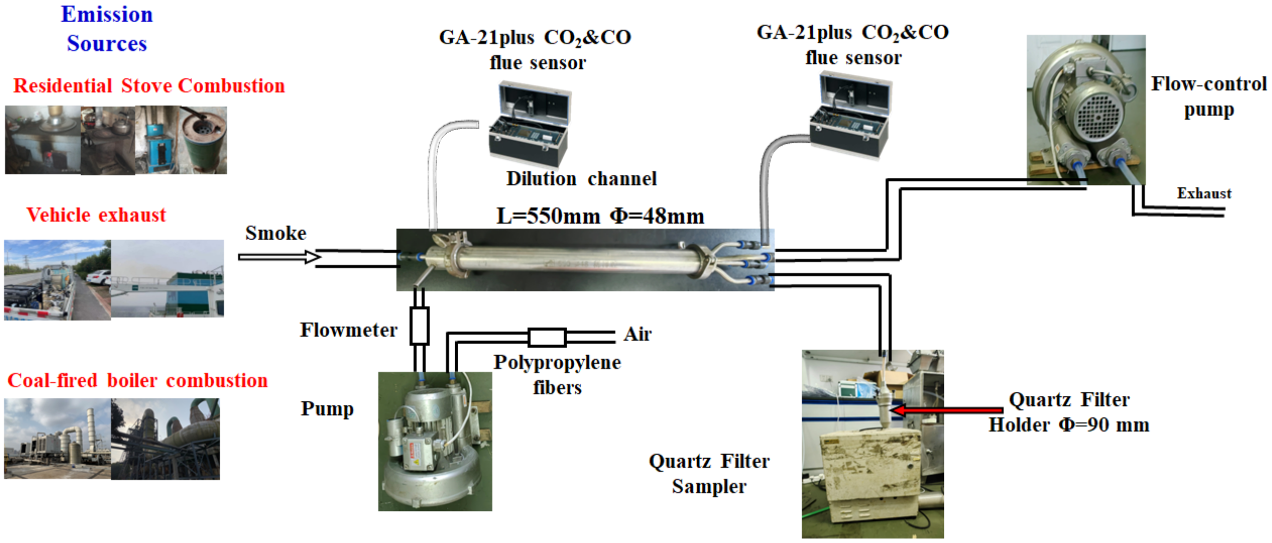

Figure S4 Sketch of the combustion and dilution sampling system of field measurements and Quartz tube furnace

References:

1. Z. Qian *et al.*, Intermediate Volatile Organic Compound Emissions from Residential Solid Fuel Combustion Based on Field Measurements in Rural China. *Environmental Science & Technology* **55**, 5689-5700 (2021).
2. Y. Zhang, Z. Yao, X. Shen, H. Liu, K. He, Chemical characterization of PM2.5 emitted from on-road heavy-duty diesel trucks in China. *Atmospheric Environment* **122**, 885-891 (2015).
3. F. Yu *et al.*, Characterization of particulate smoke and the potential chemical fingerprint of non-road construction equipment exhaust emission in China. *Science of The Total Environment* **723**, 137967 (2020).
4. Z. Liu *et al.*, Emission Characteristics and Formation Pathways of Intermediate Volatile Organic Compounds from Ocean-Going Vessels: Comparison of Engine Conditions and Fuel Types. *Environmental Science & Technology* **56**, 12917-12925 (2022).
5. F. Zhang *et al.*, Variations and characteristics of carbonaceous substances emitted from a heavy fuel oil ship engine under different operating loads. *Environmental Pollution* **284**, (2021).
6. F. Zhang *et al.*, Size-segregated characteristics of organic carbon (OC), elemental carbon (EC) and organic matter in particulate matter (PM) emitted from different types of ships in China. *Atmos. Chem. Phys.* **20**, 1549-1564 (2020).

7. Y. Han *et al.*, Existence and Formation Pathways of High- and Low-Maturity Elemental Carbon from Solid Fuel Combustion by a Time-Resolved Study. *Environmental Science & Technology* **56**, 2551-2561 (2022).

8. H. Ni *et al.*, Emission characteristics of carbonaceous particles and trace gases from open burning of crop residues in China. *Atmospheric Environment* **123**, 399-406 (2015).

9. G. Shen *et al.*, Emission Factors of Particulate Matter and Elemental Carbon for Crop Residues and Coals Burned in Typical Household Stoves in China. *Environmental Science & Technology* **44**, 7157-7162 (2010).

10. S. Tao *et al.*, Quantifying the rural residential energy transition in China from 1992 to 2012 through a representative national survey. *Nature Energy* **3**, 567-573 (2018).

11. J. Sun *et al.*, Emission factors of organic carbon and elemental carbon for residential coal and biomass fuels in China- A new database for 39 fuel-stove combinations. *Atmospheric Environment* **190**, 241-248 (2018).

12. Y. Chen *et al.*, Measurements of emission factors of PM2.5, OC, EC, and BC for household stoves of coal combustion in China. *Atmospheric Environment* **109**, 190-196 (2015).

13. Y. Hao *et al.*, Chemical characterization of PM2.5 emitted from China IV and China V light-duty vehicles in China. *Science of The Total Environment* **783**, 147101 (2021).

14. X. Li, S. Wang, L. Duan, J. Hao, Y. Nie, Carbonaceous Aerosol Emissions from Household Biofuel Combustion in China. *Environmental Science & Technology* **43**, 6076-6081 (2009).
15. G. Shen *et al.*, Field Measurement of Emission Factors of PM, EC, OC, Parent, Nitro-, and Oxy- Polycyclic Aromatic Hydrocarbons for Residential Briquette, Coal Cake, and Wood in Rural Shanxi, China. *Environmental Science & Technology* **47**, 2998-3005 (2013).
16. S. Wei *et al.*, Field measurement on the emissions of PM, OC, EC and PAHs from indoor crop straw burning in rural China. *Environmental Pollution* **184**, 18-24 (2014).
17. G. Shen *et al.*, Pollutant Emissions from Improved Coal- and Wood-Fuelled Cookstoves in Rural Households. *Environmental Science & Technology* **49**, 6590-6598 (2015).
18. W. Du *et al.*, Comparison of air pollutant emissions and household air quality in rural homes using improved wood and coal stoves. *Atmospheric Environment* **166**, 215-223 (2017).
19. W. Du *et al.*, Field-based emission measurements of biomass burning in typical Chinese built-in-place stoves. *Environmental Pollution* **242**, 1587-1597 (2018).
20. L. Zhang *et al.*, Optically Measured Black and Particulate Brown Carbon Emission Factors from Real-World Residential Combustion Predominantly Affected by Fuel Differences. *Environmental Science & Technology* **55**, 169-

178 (2021).

21. Y. Chen *et al.*, Emission Factors for Carbonaceous Particles and Polycyclic Aromatic Hydrocarbons from Residential Coal Combustion in China. *Environmental Science & Technology* **39**, 1861-1867 (2005).

22. Y. Chen *et al.*, Measurements of Black and Organic Carbon Emission Factors for Household Coal Combustion in China: Implication for Emission Reduction. *Environmental Science & Technology* **43**, 9495-9500 (2009).

23. G. Zhi *et al.*, Emission Characteristics of Carbonaceous Particles from Various Residential Coal-Stoves in China. *Environmental Science & Technology* **42**, 3310-3315 (2008).

24. G. Shen *et al.*, Comparison of carbonaceous particulate matter emission factors among different solid fuels burned in residential stoves. *Atmospheric Environment* **89**, 337-345 (2014).

25. S. D. Forestieri *et al.*, Real-Time Black Carbon Emission Factor Measurements from Light Duty Vehicles. *Environmental Science & Technology* **47**, 13104-13112 (2013).

26. R. Wang *et al.*, Global Emission of Black Carbon from Motor Vehicles from 1960 to 2006. *Environmental Science & Technology* **46**, 1278-1284 (2012).

27. Z. Peng *et al.*, Emissions from several in-use ships tested by portable emission measurement system. *Ocean Engineering* **116**, 260-267 (2016).

28. F. Zhang *et al.*, Size-segregated characteristics of OC, EC and organic matters

in PM emitted from different types of ships in China. *Atmospheric Chemistry and Physics Discussions*, 1-32 (2019).

29. F. Zhang *et al.*, Variations and characteristics of carbonaceous substances emitted from a heavy fuel oil ship engine under different operating loads. *Environmental Pollution* **284**, 117388 (2021).

30. J. C. Chow *et al.*, Source profiles for industrial, mobile, and area sources in the Big Bend Regional Aerosol Visibility and Observational study. *Chemosphere* **54**, 185-208 (2004).

31. L. W. A. Chen *et al.*, Emissions from Laboratory Combustion of Wildland Fuels: Emission Factors and Source Profiles. *Environmental Science & Technology* **41**, 4317-4325 (2007).

32. J. J. Cao *et al.*, Characterization and source apportionment of atmospheric organic and elemental carbon during fall and winter of 2003 in Xi'an, China. *Atmos. Chem. Phys.* **5**, 3127-3137 (2005).

33. Y. M. Han, J. J. Cao, S. C. Lee, K. F. Ho, Z. S. An, Different characteristics of char and soot in the atmosphere and their ratio as an indicator for source identification in Xi'an, China. *Atmos. Chem. Phys.* **10**, 595-607 (2010).

34. K. He *et al.*, Saccharides Emissions from Biomass and Coal Burning in Northwest China and Their Application in Source Contribution Estimation. *Atmosphere* **12**, (2021).

35. S. C. L. J.J. Cao, K.F. Ho , Kochy Fung , Judith C. Chow，John G. Watson,

Characterization of Roadside Fine Particulate Carbon and its Eight Fractions in Hong Kong. *Aerosol and Air Quality Research,* **6**, 106-122 (2006).

36. R. Wang *et al.*, Trend in Global Black Carbon Emissions from 1960 to 2007. *Environmental Science & Technology* **48**, 6780-6787 (2014).

37. H. Xu *et al.*, Updated Global Black Carbon Emissions from 1960 to 2017: Improvements, Trends, and Drivers. *Environmental Science & Technology* **55**, 7869-7879 (2021).

38. Y. Fang *et al.*, Spatiotemporal Trends of Elemental Carbon and Char/Soot Ratios in Five Sediment Cores from Eastern China Marginal Seas: Indicators of Anthropogenic Activities and Transport Patterns. *Environmental Science & Technology* **52**, 9704-9712 (2018).
